# Low self-reported stress despite immune-physiological changes in paramedics during rescue operations

**DOI:** 10.17179/excli2021-3617

**Published:** 2021-04-16

**Authors:** Corinna Peifer, Vera Hagemann, Maren Claus, Mauro F. Larra, Fabienne Aust, Marvin Kühn, Monika Owczarek, Peter Bröde, Marlene Pacharra, Holger Steffens, Carsten Watzl, Edmund Wascher, Silvia Capellino

**Affiliations:** 1 University of Lübeck, Department of Psychology, Lübeck, Germany; 2 University of Bremen, Faculty of Business Studies and Economics, Bremen, Germany; 3 Leibniz Research Centre for Working Environment and Human Factors (IfADo), Department of Immunology, Dortmund, Germany; 4 IfADo- Leibniz Research Centre for Working Environment and Human Factors (IfADo), Department of Ergonomics, Dortmund, Germany; 5 Ruhr University Bochum, Faculty of Psychology, Bochum, Germany; 6 MSH Medical School Hamburg, University of Applied Sciences and Medical University, Hamburg, Germany; 7 Arbeiter-Samariter-Bund, Dortmund, Germany

**Keywords:** stress, burnout, coping, immune system, cortisol, heart rate variability, work/psychological demands

## Abstract

Despite the high stress levels, paramedics seem to ignore or even negate the stress. This can be detrimental and lead to stress-related diseases. Therefore, we investigated the divergence between physiological and psychological stress responses of paramedics. Participants were 16 paramedics and 17 white-collar workers. We assessed psychological stress parameters, cortisol awakening response (CAR), and quantified immune parameters. In paramedics, electrocardiogram (ECG) was measured during one complete 24-hour shift. Our results revealed that CAR was higher in paramedics compared to controls. An alteration of immune parameters was observed even during days of free time. Also, ECG recordings showed acute stress in paramedics during rescue situations. Questionnaires revealed that rescue-service specific stressors affect psychological outcomes. However, paramedics reported significantly less mental stress and higher levels of depersonalization than controls. Taken together, our results suggest higher stress in paramedics compared to controls. However, paramedics negate their daily stress. Our findings underline therefore the importance to develop stress-management interventions for paramedics including sensitization for their stress reactions.

## Introduction

Employees working in so called medium or high risk professions, such as emergency workers, are often exposed to acute stressors such as the confrontation with suffering persons, exposure to violence and death, low perceived appreciation of their work, as well as disruption of the day/night rhythm due to shift work. The continuous confrontation with stressors can lead to physical and mental stress signs, such as headache, high blood pressure, impatience and defensiveness. A recent study reveals a higher rate of sick leave in nurses from the emergency department compared to other hospital staff, despite a general healthy psychosocial work condition was reported (Rugless and Taylor, 2011[44]). Another study reported over 19 % of burnout in emergency department professionals, with higher incidence in paramedics compared to physicians (Durand et al., 2019[19]). Exposure to major disasters such as airplane crashes and earthquakes leads to serious mental disturbances, such as post-traumatic-stress-disorder (PTSD). Such events are luckily very rare in rescue emergency operations. However, it was shown that a substantial amount of emergency workers (21 %) who were *not* exposed to major disasters also experienced symptoms of PTSD (Clohessy and Ehlers, 1999[13]), and it was suggested that frequently occurring critical incidents can be just as stressful for emergency workers as disaster work (Marmar et al., 1996[36]). Of interest, there are also studies showing that paramedics declare to be *less* stressed compared to other professionals (Regehr et al., 2002[43]; Janka and Duschek, 2018[31]), which contradicts the previous studies.

Accordingly, smaller but frequently recurring stressors may lead to major psychological changes in emergency workers and should not be underestimated (van der Ploeg and Kleber, 2003[50]). Moreover, there seems to be a discrepancy between subjectively reported stress and long-term outcomes such as increased burnout in paramedics (Oldehinkel et al., 2011[41]; Hellhammer and Schubert, 2012[28]). Our study thus aims for a better understanding of daily stressors and stress outcomes in paramedics using a broad measurement approach. Besides measuring subjective psychological stress markers, we included physiological stress markers from the immune system, the endocrine system, and the autonomous nervous system. 

It has already been demonstrated that stress can directly influence the immune system and also alter many physiological functions, such as heart rate and cortisol release (Glaser and Kiecolt-Glaser, 2005[23]; Fries et al., 2009[22]). Immune activation in response to stressors serves to support the requirements of the “fight or flight” response, and to get the body ready for possible injuries and thus infections. It is described that shortly after the exposure to acute stressors immune cells are recruited into the blood stream (Elsenbruch et al., 2006[20]), and the amount of proinflammatory cytokines is increased (Segerstrom and Miller, 2004[47]; Maydych et al., 2018[39]). These results indicate that the immune system is very effective in the acute response to stressors.

However, the stressors of contemporary society often do not require or even do not allow behavioral “fight or flight”, and the response of the immune system during chronic psychological stress substantially exceeds physiological requirements (Cacioppo et al., 1998[7]). It is in fact reported that the amount of proinflammatory cytokines is elevated in the blood during chronic stress, but the ability of the immune cells to respond and fight is reduced. It is, for example, demonstrated that chronic stress suppresses or dysregulates innate and adaptive immune responses by altering the Type 1-Type 2 cytokine balance (Segerstrom and Miller, 2004[47]; Dhabhar, 2014[17]). On the other hand, during chronic stress there is a reduction of the number of cytotoxic T cells and of natural killer (NK) cells (Glaser et al., 1986[25]), a lower lymphocyte proliferation in response to specific mitogens (Halvorsen and Vassend, 1987[27]; Dobbin et al., 1991[18]), as well as decreased percentages of CD4 helper T cells and CD8 cytotoxic T cells (Glaser et al., 1985[24]), thus suggesting that the immune system is exhausted due to the stress-related chronic alert state.

It is important to point out that the same stressor can have profoundly different effects on the immune system activation across individuals or in different life circumstances within the same individuals (summarized in Cacioppo et al., 1998[7]; Segerstrom and Miller, 2004[47]). Therefore, the amplitude of the effects described above can strongly vary within and between subjects.

Acute stress also activates the hypothalamic-pituitary-adrenal-axis (HPA) as well as the sympathetic nervous and adrenomedullary systems. Enhanced sympathetic tone increases cardiovascular arousal, causing rises in heart rate (HR). Typically, such stress-induced alternations are quickly counter-regulated by the parasympathetic nervous system. Measuring variations in HR over longer time-periods therefore provides a method to trace stress exposure and regulation by physiological means (Chandola et al., 2010[9]). Indeed, previous studies assessing heart rate variability (HRV) parameters during work shifts have reported significant negative associations of dominant parasympathetic activation with experienced stress (Collins and Karasek, 2010[14]; Clays et al., 2011[12]; Hernandez-Gaytan et al., 2013[30]; Tonello et al., 2014[49]; Borchini et al., 2015[4]).

The HPA axis is another highly stress-responsive system and persistent alterations of its activity due to chronic stress are known to be reflected in the cortisol awakening response (CAR). The CAR denotes a considerable increase of cortisol concentrations due to awakening which was found to be altered by work related stress (for review see Fries et al., 2009[22]).

So far, comprehensive studies investigating stress-related physiological changes in paramedics during and after rescue operations are scarce. While increases in heart rate during emergency call responses are established (Backe et al., 2009[2]; Karlsson et al., 2011[32]), studies investigating the HRV pattern reported changes only in paramedics with many health complaints (Aasa et al., 2006[1]). Moreover, studies indicate higher morning cortisol on days with emergency calls in some paramedics compared to workfree days or days working with non-emergency patients (Aasa et al., 2006[1]; Backe et al., 2009[2]). 

Based on this knowledge, it is plausible that recurrent stress situations also alter physical health and wellbeing of emergency workers. However, estimating the impact of these changes (van der Ploeg and Kleber, 2003[50]) seems difficult, as an understanding about the interplay of immunological, psychological and physiological changes in paramedics is lacking. Aim of this study was therefore to analyze and compare a large spectrum of immunological, psychological and physiological parameters in paramedics. A multi-method approach was taken: First, a cross-sectional study was used to look at outcomes of rescue-service specific stressors. Second, we compared self-reported stress and cortisol awakening response between paramedics and white-collar workers. Third, immunological data of paramedics was analyzed after a day of work and compared to immunological values after at least one day of free time. As reference for immunological parameters, results from a healthy cohort from another study were used. Last, the impact of a 24 h shift on paramedics' immediate psychological wellbeing and the autonomous nervous system was tracked. 

## Methods

### Participants

Participants were 16 ambulance service paramedics from four different rescue stations of the non-profit aid and rescue organization ASB [“Arbeiter-Samariter-Bund”, Workers' Samaritan Federation] and 17 age- and sex-matched white-collar workers as control. The study has been performed in accordance with the ethical standards as laid down in the 1964 Declaration of Helsinki and its later amendments and was revised and approved by the ethical committee (IfADo 2017/128/2017-10-07). Each subject gave written informed consent for participation in the study after the nature and possible consequences of the study had been fully explained. Participation to the study was completely voluntary and had no impact on the operational procedures at work. As reference for immune data, results from paramedics were compared to an age- and sex-matched healthy cohort from another study. Of interest, younger paramedics were more prone to participate to the study than older colleagues.

### Study design

**Figure 1:**
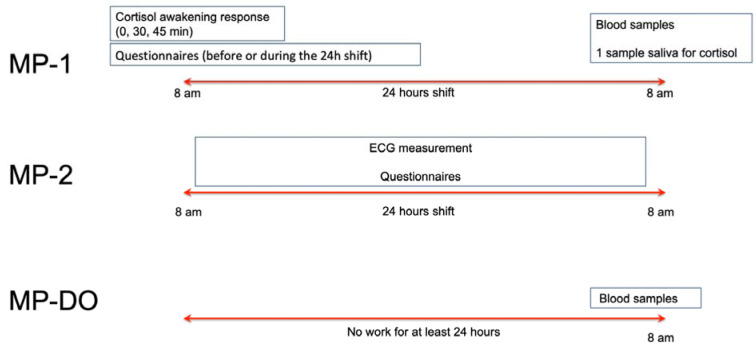
Study design. Schematic representation of the study design for paramedics. MP = measurement point; DO = day off. Each measurement point represents one day of sample collection (for MP1 and MP2 this means a work shift from 8 am to 8 am the day after, for MP-DO one day off) in which samples and/or data were collected. MP-DO represents one resting day or vacation day of at least 24 hours. For controls, cortisol awakening response, questionnaires and blood samples were collected at the same day time and in the same way as in paramedics. Control subjects did not have a 24h shift.

A scheme of the study design is shown in Figure 1(Fig. 1). *Measurement point 1 (MP-1):* All paramedics had a 24 h shift and at least two days off prior to the shift. Paramedics neither work in fixed teams nor are they allocated to a fixed rescue station leading to new team composition at every rescue station for every shift. Cortisol awakening response of paramedics was measured immediately after awakening at the day of the work shift (shift started at 8 am) and one more cortisol measure was taken at the end of the work shift (8 am, 24 hours later). Due to the irregular sleep phases during the 24 h shift, a cortisol awakening response measurement at the end of the shift was not evaluated. At the end of the shift, blood samples were collected for the measurement of immune parameters, as described below. In the day prior to the shift, paramedics completed the questionnaires described below. Paramedics were asked not to talk about the content of the questionnaires to other colleagues.

For the control group, the cortisol awakening response of the white-collar workers and the cortisol level 24 hours later were collected at the same time points and in the same way as those of the paramedics. Also, the samples were collected in the same time of the year for both groups. White-collar control subjects filled in the psychological questionnaires the day prior to cortisol measurements. For immunological data, results from age- and sex-matched healthy donors from a previous study were used as reference. In both cohorts, blood samples were collected 8-10 am, in order to minimize any circadian difference. 

To find out if immunological parameters are eventually altered in paramedics only acutely after the shift or if the effects are long-lasting, we collected blood samples from the paramedics after at least 24 hours of break (MP-DO) at the same time point as the other blood samples (8 am).

*Measurement point 2 (MP-2):* To study possible effects of emergency operations on stress and sympathetic tone, a 24 hours ECG was acquired during another 24 h shift (MP-2). Paramedics had approximately 2-3 weeks of regular work between MP-1 and MP-2.

### Self-report questionnaires:

Participants were asked to complete self-report questionnaires in order to access perceived stress during work and leisure time as well as the handling of these stressors. In the following we introduce the questionnaires that are relevant to this study. The questionnaire was 15 pages long and participants needed 35 minutes to fill it out. More detailed information about the questionnaires including values of coefficient alpha as test score reliability coefficients (Cronbach and Warrington, 1951[15]) are provided in the Supplementary Material. 

**Stress from rescue service specific stressors** was measured with the “Stress during rescue service mission Scale” (SIRE) (Hagemann and Holtz, 2016[26]) on a 5-point rating scale. The SIRE refers specifically to stressors during rescue missions. It consists of six subscales named “competence”, “violence”, “not self-inflicted external factors”, “emergency operation”, “migration”, and “unpredictable missions”. 7 items were added to the scale, complementing the subscales “interruptions”, “colleagues”, “alteration” and “material”. The SIRE was only filled in by the paramedics.

**Mental Stress** was assessed with the 8-item Irritation Scale (Mohr et al., 2005[40]) on a 7-point rating scale. The scale consists of the two dimensions “emotional irritation” and “cognitive irritation”. 

**Subjective strain** was assessed with the German version (Fliege et al., 2001[21]) of the Perceived Stress Questionnaire (Levenstein et al., 1993[35]) (PSQ) on a 4-point rating scale. The questionnaire includes the four subscales “tension”, “demands”, “joy”, and “worries”.

**Chronic stress** was assessed with the Trier Inventory of Chronic Stress (TICS) (Schulz et al., 2004[46]). The inventory consists of nine subscales named “work overload”, “social overload”, “pressure to perform”, “work discontent”, “excessive demands at work”, “lack of social recognition”, “social tensions”, “social isolation” and “chronic worrying”. The items were rated on a 5-point rating scale.

**Detachment** was measured with the Recovery Experience Questionnaire (Sonnentag and Fritz, 2007[48]) on a 5-point rating scale. The questionnaire consists of four subscales named “psychological detachment”, “relaxation”, “mastery” and “control”. 

**Coping Strategies** were measured with a 4-point rating scale based on the German Brief (Knoll et al., 2005[33]) COPE questionnaire (Coping Orientation to Problems Experienced) (Carver et al., 1989[8]). The subscales are “acceptance”, “use of instrumental support”, “denial”, “positive reframing”, “behavioural disengagement”, “substance use”, “self-distraction”, “use of emotional support”, “humour”, “active coping”, “venting”, “planning”, “self-blame”, and “religion”. To specify the subscale “use of instrumental support” we divided between professional advice, private advice and advices from colleagues. 

**Burnout risk** was measured with the German version (Büssing and Perrar, 1992[6]) of the Maslach Burnout Inventory (Maslach and Jackson, 1981[37]) (MBI). The two subscales “emotional exhaustion” (EE) and “depersonalisation” (DE) were used. The items were measured on a 6-point rating scale.

**Positive and Negative Affect** were measured with a German version (Krohne et al., 1996[34]) of the Positive and Negative Affect Schedule (PANAS) (Watson et al., 1988[51]) using a 5-point rating scale, to access the “positive affect” and “negative affect”.

**Work-related flow experience** was assessed with the 13-item Work-Related Flow Inventory (WOLF) (Bakker, 2008[3]) on a 7-point rating scale*. *The inventory consists of three subscales named “absorption”, “work enjoyment”, and “intrinsic work motivation”.

Relationships between rescue-service specific stressors and psychological outcomes were evaluated with Pearson correlations. In case of violation of the conditions of normal distribution (skewness > |2| or kurtosis > |7|) (Curran et al., 1996[16]; Ryu, 2011[45]) we used the Spearman correlation. In a further analysis, *t*-tests were calculated in order to analyze significant differences between the paramedics and the white-collar controls. If the variances were unequal, we used the *t*-test for unequal variances (Welch's *t*-test). In case of violation of the conditions of normal distribution (skewness > |2| or kurtosis > |7|) (Curran et al., 1996[16]; Ryu, 2011[45]) we used the Mann-Whitney-U-test.

### Determination of cortisol levels

Saliva samples for determination of cortisol awakening response (CAR) were taken immediately after wake up, and 30 min and 45 min after awakening in control participants and paramedics. An additional cortisol sample was taken 24 h later in both groups corresponding to the end of the shift for the paramedics. Saliva was sampled using the Salivette® Cortisol with synthetic swab (Sarstedt, Germany). The participants were instructed to place the swab in their mouth for one minute with gentle movement. Samples were then stored at 4 °C and centrifuged within 24 hours. Saliva samples were then stored at -80 °C until analysis. 

**Figure 2:**
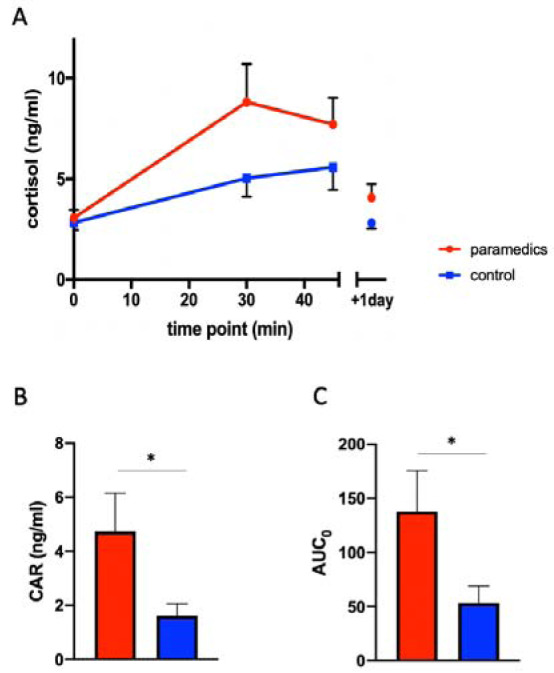
Cortisol levels are higher in paramedics compared to control group. A: Cortisol awakening response in the paramedics (red, n= 15) compared to white-collar controls (blue, n= 16). B: CAR levels measured in paramedics (red) and white-collar controls (blue). CAR = difference between the maximal cortisol level and basal cortisol level, calculated for each subject. C: Area under the curve (AUC_0_) measured in paramedics (red) and white-collar controls (blue). AUC_0_ = area under the curve set to baseline level. Results represent mean ± SEM. Paired t-test. * = p 0.05.

Cortisol levels were measured in saliva by ELISA as described by the manufacturer (IBL International, Hamburg, Germany). The maximal level of cortisol reached within the 45 min after awakening in comparison to the cortisol level at time 0 min was evaluated as cortisol awakening response (CAR). CAR values and the area under the curve (AUC_0_, as described in Figure 2B(Fig. 2)) of paramedics and control group were compared using an unpaired *t*-test. P values <0.05 were considered significant.

### Analysis of immunological parameters

#### Absolute cell count

To determine the absolute number of leukocyte populations by multicolor flow cytometry, 50 µl fresh heparinized blood sample were added to TruCount tubes (BD Biosciences, Heidelberg, Germany) and incubated for 20 min at RT with the indicated antibodies (Supplementary Table 2). Samples were subjected to erythrocyte lysis using FACS Lysing Solution (BD Biosciences), and were measured on a BD LSRFortessa (BD Biosciences). Data were analyzed using FlowJo Software (FlowJo LLC, USA). Absolute cell number in blood samples was calculated as:

(# events in cell gate)/(# events in bead gate)*(# beads per test)/(test volume) = cells per µl blood

#### Multicolor flow cytometry

Three different panels were set up to analyze the samples for (1) general overview of lymphocytes and monocytes, (2) memory and homing markers of natural killer (NK) and T cells, (3) activation and memory markers of NK and T cells. All antibodies were individually titrated to determine the optimal dilution. All antibodies and dilutions are listed in Supplementary Table 2. Gating strategy is shown in Supplementary Figure 1. Detailed informations regarding the various markers and gated lymphocyte subpopulations are provided by Claus et al. (2016[11]).

Peripheral blood mononuclear cells (PBMC) were used immediately after thawing and were kept on ice during the staining procedure. For each panel, 0.2 x 10^6^ cells were stained with the indicated antibody cocktails for 20 min at 4 °C in the dark and then washed with FACS buffer (PBS / 2 % FCS). Cells were resuspended in 150 µl FACS buffer and kept on ice until analysis at the same day on a BD LSRFortessa. Data were analyzed using the FlowJo software (FlowJo LLC, USA). For the statistical analysis, Kruskal-Wallis test with Dunn's post-test was performed. 

#### Cytokine assays

Cytokine levels in serum were measured using the LEGENDplex Human Inflammation Panel (13-plex) (BioLegend) according to the manufacturer's instructions with slight modifications (Maydych et al., 2018[39]).

Briefly, 10 µl serum or standard was added to a V-bottom 96 well plate and mixed with 30 µl assay buffer, 10 µl beads and 10 µl biotinylated detection antibody mix and incubated for 2 h at RT on a shaker at 600 rpm. Then, 10 µl PE-conjugated Streptavidin was added, followed by an additional incubation for 30 min at RT on a shaker at 600 rpm. After 2 washes with wash buffer (provided in the kit), PE fluorescence intensity of the beads was measured on a LSRFortessa flow cytometer (BD BioSciences). Beads populations were identified by FSc/SSc features and fluorescence intensity in the APC channel. Approx. 400 beads per analyte were acquired. Data were analyzed using the LEGENDplex^TM^ Data Analysis Software (VigeneTech). All samples were analyzed at the same day to avoid inter-assay variation. According to the manufacturer, intra-assay variation is between 3 and 9 % CV. Minimum detectable concentration is between 0.6 pg/ml for IL-12 and 1.9 pg/ml for IL-17A. The statistical analysis of IL-6 and IL-18 was performed with the Kruskal-Wallis test with Dunn's post test. For IL-17 and IL-23, the Χ^2^ test was used. Differences between samples were not significant for IL-1β, IFN-α, IFN-γ, TNF-α, MCP-1, IL-8, IL-10, and IL-12p70. Serum concentration of IL-33 was below detection limit.

### ECG recording and analysis

To trace stress exposure in paramedics over the shift by physiological means we aimed in this study to continuously acquire ECG during the complete 24 h shift. Following previous findings, we expected to find higher HR and reduced vagal tone during emergency vs. resting periods and corresponding associations with self-reported stress and morning cortisol as a measure of HPA-axis regulation.

A single-channel ECG was continuously recorded with the mobile eMotion 360° Faros device (Mega Electronics, New Brunswick, NJ) during a 24 h shift at measurement point 2, i.e. 2-3 weeks after measurement point 1. The device was attached to a flexible belt with integrated electrodes and placed directly beneath participants' breast. This cablefree system allows for a non-interfering and comfortable measurement of continuous ECG data with low artifact contamination over longer periods of time. The ECG signal was stored to the device with a sampling rate of 1000 Hz at 16 bit resolution. Beat detection and calculation of time- and frequency domain HRV parameters were performed offline using HRV-Scanner software (Biosign, Ottenhofen, Germany), as was artifact control. Identification of resting and emergency call phases during the 24 h shift in the ECG data was based on a provided, written documentation by the paramedics about the time and duration of the emergency calls.

Sine rhythm was confirmed and epochs with artifacts rejected via Poincaré-based filtering followed by visual inspection. The percentage of rejected data epochs was below 5 % in all subjects. Heart rate, RMSSD and normalized LF/HF-Ratio were calculated for the whole 24 h shift as well as separately for emergency calls and resting phases between emergencies. Resting and emergency phases were then averaged separately with each epoch weighted by its length. Frequency bands used for extraction of high and low frequency spectral power ranged from 0.15 - 0.40 Hz and 0.04 - 0.15 Hz, respectively.

## Results

We enrolled 16 paramedics (69 % male, mean age 32.4, age range 21-55) and 17 age- and sex-matched white-collar workers as controls (71 % male, mean age 33.9, age range 18-54). For immune data, results from paramedics were compared to an age- and sex-matched healthy cohort from another study (n=16, 69 % male, mean age 32.3, age range 20-54). Due to outlier removal, the precise n-number used for each analysis is shown in the specific pictures and “results” sections.

### Correlations between rescue-service specific stressors and psychological consequences

Pearson correlations were calculated between stress from rescue service specific stressors (SIRE) and psychosocial consequences such as stress, positive and negative affect, and work-related flow experience. The results are shown in Table 1(Tab. 1). We found that high values for experiencing rescue service specific stressors were significantly related to an increase in worries, social isolation, irritation, emotional irritation and negative affect and a decrease in work-related flow, work enjoyment, joy and psychological detachment. Furthermore, the experience of rescue service specific stressors was positively associated with the use of coping strategies, especially self-distraction, denial, and religion.

There were a wide range of correlations between the subscales of the SIRE and the different psychological measurements. For the sake of brevity, we just described a few exemplary relationships here. 

Particularly stress from the stressor *unpredictable missions* had a high mean value and showed interesting correlations with our outcome variables: it was related to increased work discontent, chronic worrying, irritation, and negative affect, and to decreased work enjoyment. Regarding coping strategies, high values of stress due to unpredictable missions was significantly related to increased substance use and use of instrumental support, but to decreased acceptance (Table 1(Tab. 1)). 

These results demonstrate the significant effects of experiencing stress due to rescue-service specific stressors during a shift on increased stress-related experiences, reduced work enjoyment and the application of problematic coping mechanisms.

### Differences between paramedics and white-collar controls

Referring to Table 2(Tab. 2) we found significant differences between paramedics and white-collar controls of mental stress, particularly on the subscale cognitive irritation, with paramedics reporting significantly *less* mental stress than controls. Furthermore, we found differences in the coping strategies positive reframing and denial, which paramedics reported to use more frequently. In addition, paramedics showed significantly higher levels on the burnout-subscale 'depersonalisation'. 

### Paramedics have higher cortisol levels compared to controls

Cortisol awakening response levels were higher in the paramedics compared to white-collar controls (Figure 2A(Fig. 2)). Despite the moderate sample size (15 paramedics and 16 controls due to outliers removal), the differences in the cortisol awakening response (CAR) and the total cortisol level between paramedics and controls were significant (Figure 2B-C(Fig. 2)). Cortisol levels after 24 h were slightly higher but not significantly different compared to basal cortisol levels measured the day before in paramedics, whereas cortisol levels were almost identical to the day before in the white-collar controls (Figure 2A(Fig. 2)). Basal cortisol levels of the paramedics prior the 24 hours shift were similar to those of white-collar controls (Figure 2A(Fig. 2)), suggesting that the alterations of cortisol levels in paramedics are only elevated during work and not chronically altered.

### Paramedics show altered immunological parameters 

**Figure 3:**
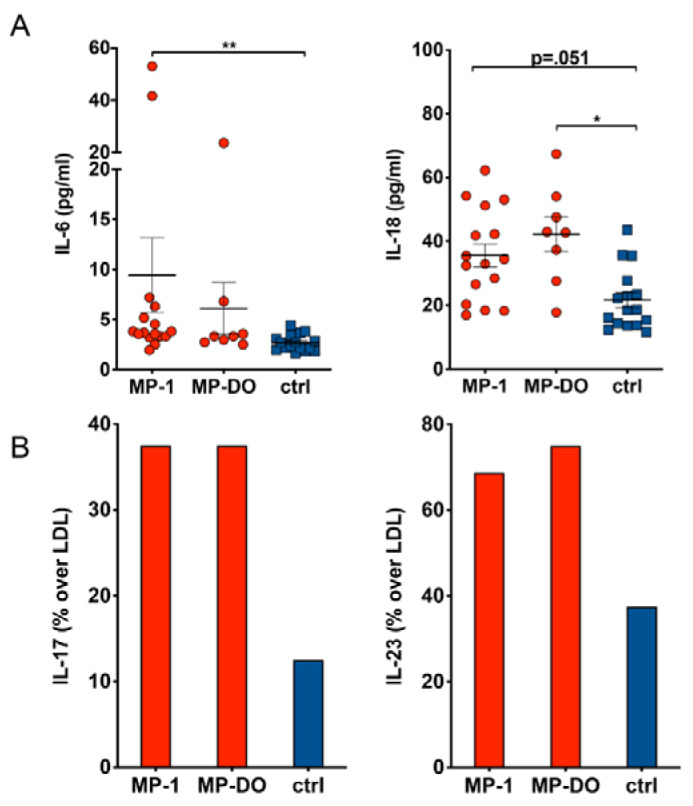
Cytokine concentration in serum. Cytokine concentration was determined in serum samples from paramedics after a 24 h shift (MP-1) or after at least 24 h rest (MP-DO). Samples from healthy subjects served as controls (ctrl). A: IL-6 and IL-18 serum concentrations are slightly increased in paramedics compared to controls. (Kruskal-Wallis test with Dunn's post test. * p<.05, ** p<.01). B: Serum concentration of IL-17 and IL-23 is more frequently above lower detection limit (LDL) of the assay in paramedics compared to controls. Due to the low N number the differences do not reach statistical significance (Χ^2^ test).

We found significantly elevated levels of IL-6 and IL-18 in serum of paramedics compared to healthy subjects (Figure 3A(Fig. 3)). This increase was independent of possible acute stress during work, as we found similarly elevated cytokine levels also after at least 24 h of free time. Further, serum concentration of IL-17 and IL-23 was more frequently above the lower detection limit (LDL) of the assay, indicating higher serum levels also of these cytokines in paramedics (Figure 3B(Fig. 3)). 

**Figure 4:**
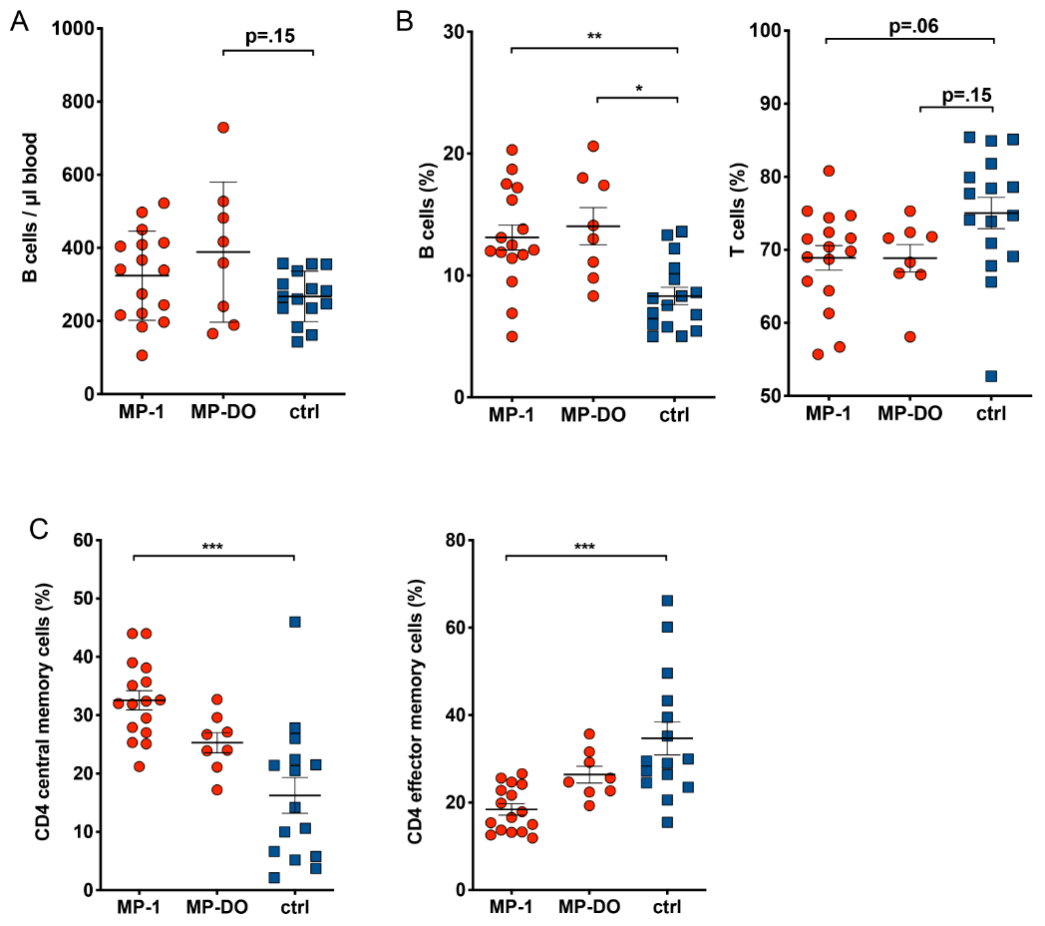
Distribution of lymphocyte subpopulations. A: Percentage of T and B cells was determined by flow cytometry. T cells were identified as CD3+ and CD56-, B cells were CD19+ and CD3-. B: Absolute number of leukocytes was determined by flow cytometry using BD TruCount tubes. B cells were CD19+ and CD3-. Absolute numbers of B cells and percentages of T cells and B cells in paramedics are changed compared to control samples. C: Percentage of naive and memory T cell subsets was measured by flow cytometry. In CD4 T cells, the ratio between central (CD45RA-, CCR7+) and effector (CD45RA-, CCR7-) memory cells is inverted compared to control samples. (Kruskal-Wallis test with Dunn's post test. * p<.05, ** p<.01). MP-1: samples from paramedics after 24 h shift; MP-DO: samples from paramedics after at least 24h rest; ctrl: samples from healthy subjects.

Analyzing the absolute numbers of lymphocytes, monocytes and granulocytes in fresh blood samples, we found that the number of B cells was slightly increased in paramedics (Figure 4A(Fig. 4)). This effect was stable, as we found a comparable increase in B cell numbers also after at least 24 h of free time.

The changes in absolute B cell numbers were paralleled by a shift of the B-to-T cell ratio within peripheral blood lymphocytes towards significantly higher B cell percentages in paramedics (Figure 4B(Fig. 4)). 

Multicolor immunophenotyping by flow cytometry allowed us to perform a more detailed analysis of PBMC subpopulations. We developed three panels to analyze the most common lymphocyte subpopulations in clinically healthy individuals. We were particularly interested in alterations in subset composition and the expression of markers for activation or maturation of lymphocytes in response to chronic stress. Detailed information about the analyzed markers and lymphocyte subpopulations is provided by Claus et al. (2016[11]). This analysis revealed that in addition to the lower T cell percentages, there was also a shift among the different memory T cell subpopulations. In paramedics, we observed a significant reduction in the percentages of CD4+ effector memory T cells and, accordingly, an increase in CD4+ central memory T cells (Figure 4C(Fig. 4)).

### Percentage of NK cells correlates with stress from rescue service specific stressors

**Figure 5:**
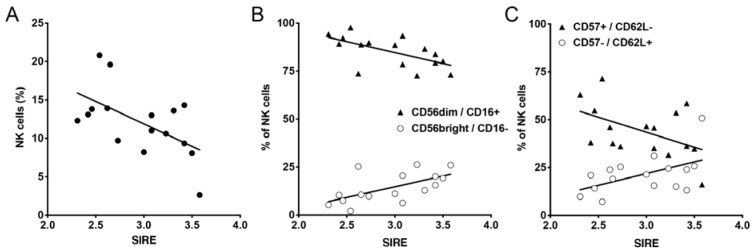
Correlation of paramedics NK cell subsets with stress from rescue service specific stressors (SIRE). A: NK cells (CD56+ / CD3- lymphocytes) were analyzed by flow cytometry and percentage of NK cells was correlated with SIRE score. B: Maturation state of NK cells was defined by CD56bright / CD16- (less mature) and CD56dim / CD16+ (more mature) and correlated with SIRE scores. C: Maturation state of NK cells was defined by CD57- /CD62L+ (less mature) and CD57+ / CD62L- (more mature) and correlated with SIRE scores. Pearson correlation was performed and showed statistically significant correlation for almost all the observed parameters (A: p = 0.02; B: p = 0.011 for CD56 bright/CD16- and p = 0.012 for CD56dim/CD16+ NK cells; C: p = 0.042 for CD57-/CD62L+ and p = 0.056 for CD57+/CD62L- NK cells).

NK cell percentages were lower in paramedics who showed higher scores of stress from rescue service specific stressors (Figure 5A(Fig. 5)). A more detailed analysis revealed an increase of the less mature CD56bright / CD16- NK cell subset with increasing rescue service specific stress scores and a negative correlation of rescue service specific stress scores with the percentage of more mature CD56dim / CD16+ NK cells (Figure 5B(Fig. 5)). A similar shift towards less mature NK cell subsets was found when we analyzed the distribution of more mature CD57+ / CD62L- and less mature CD57- / CD62L+ NK cells (Figure 5C(Fig. 5)). Taken together, these results indicate a reduced amount of mature NK cells in paramedics with higher rescue service specific stress. Similar results were also observed for the stress due to the stressors “violence” and “unpredictable missions” (Supplementary Figure 2).

### HR and HRV differ between emergency and resting phases

**Figure 6:**
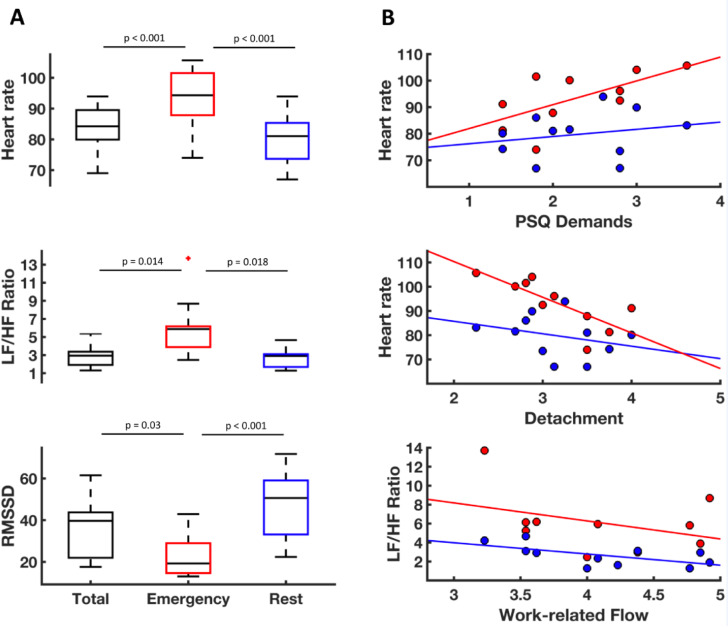
Effects of the 24 h shift on heart rate and heart rate variability. A: Heart rate, ratio of high and low frequency heart rate variability (LF/HF Ratio) and root mean square of successive differences (RMSSD) during the complete 24 h shift (“total”, black boxes) as well separately during emergency calls (“emergency”, red boxes) and in-between emergencies (“rest”, blue boxes). Heart rate was significantly elevated during emergency calls compared to rest and 24 h recording as was LF/HF Ratio. RMSSD was lower during emergency compared to rest and 24 h total. B: Associations of HRV indices during rest (blue) and emergency (red) with psychological stress questionnaires. Heart rate during emergency was positively correlated to the PSQ-subscale “demands” (r = .65, p = .044) and negatively correlated to the detachment as measured with the Recovery Experience Questionnaire (r = -.77, p = .010). Flow was negatively correlated with the LF/HF ratio under rest (r = -.62, p = .043)

ECG data were available from 11 participants. Separate repeated-measures ANOVAs were conducted on heart rate, RMSSD and LF/HF-Ratio with phase (emergency, rest, total 24 h) as within-subject factor revealing a main effect of phase for all dependent variables. Heart rate was significantly elevated during emergency calls compared to rest and 24 h recording as was LF/HF. By contrast, RMSSD was lower during emergency compared to rest and 24 h total (Figure 6A(Fig. 6)). These results demonstrate that acute stress during the shift is reflected in the ECG recordings in a meaningful way, with increases in heart rate, parasympathetic withdrawal (lower RMSSD) and enhanced sympathetic tone (increased LF/HF ratio) observable during emergency situations.

### HRV and HR during a 24 h shift are correlated with psychological variables in paramedics

We computed Pearson correlations between HRV indices 'psychological stress' and 'work questionnaires'. Some significant correlations of psychological variables with HR and HRV parameters could be observed. In particular, heart rate during emergency showed a strong positive relation to the PSQ-subscale “demands”. The opposite relation was observed for detachment (measured with the Recovery Experience Questionnaire), which was negatively correlated to emergency heart rate, i.e. higher heart rate during emergency was related to reduced detachment from work. Furthermore, flow in total and particularly work enjoyment was negatively correlated with the LF/HF ratio under rest indicating that a lower LF/HF ratio under rest was associated with higher flow and work enjoyment (Figure 6B(Fig. 6)).

## Discussion

Stress from rescue service specific stressors was associated with increased psychosocial stress - especially emotional irritation -, increased negative affect, and reduced work enjoyment. Especially stress from the category 'unpredictable missions' was associated with increases in chronic worrying and work discontent, presumably resulting from the aversive requirements (Schulz et al., 2004[46]).

Also, we found associations of stress from unpredictable missions with alcohol and drug consumptions as coping mechanisms - which is peculiar and alarming. 

When comparing paramedics with healthy controls, our results reveal that paramedics show *lower* stress values than the white-collar workers. However, when looking at the cortisol-awakening response (CAR), a marker of current or chronic stressful circumstances, we found just the opposite pattern: paramedics show *a more pronounced CAR before the acute work situation, *which could be a sign of increased stress levels compared to the white-collar workers. A possible explanation for these seemingly incompatible results could be that paramedics tend to deny the experience of psychological strain to keep the picture of the “strong paramedic” upright - even without being aware of this (Regehr et al., 2002[43]). Also, dissociations between psychological and physiological stress markers have been found before (Oldehinkel et al., 2011[41]; Hellhammer and Schubert, 2012[28]). Accordingly, our results confirm our prior assumption that psychological stress markers are not increased in paramedics compared to controls, while the physiological markers tell another, unbiased picture. 

Interestingly, previous studies indicated that higher morning cortisol in paramedics may be restricted to days actually working emergency calls, suggesting that these physiological changes may be transient (Aasa et al., 2006[1]; Backe et al., 2009[2]). 

However, our results also show a general alteration of specific immune parameters such as the amount of proinflammatory cytokines and the percentage of immune cell subpopulations in paramedics, even during days of free time. These immunological alterations are well described in chronic stress situations (for reviews, see Herbert and Cohen, 1993[29]; Yang and Glaser, 2002[52]; Segerstrom and Miller, 2004[47]) and suggest that paramedics are subjected to stress-related immunological changes. The amount of cytokine measured is not pathological, but the significant higher levels even in the small sample size reveals an alteration of the immune response due to typical daily stress experienced by paramedics. Also, we found a lower level of mature NK cells, which was previously demonstrated to correlate with high stress levels. Similar results were also observed in a previous study (Maydych et al., 2017[38]). The reason for the reduced amount of mature NK cells during stress is not clear so far, but our hypothesis is that mature immune cells migrate into lymphnodes instead of circulating in the blood. Taken together, the alteration of immune parameters is a good indicator of a chronic stress situation, even if the paramedics deny the stress.

The analysis of HRV parameters obtained during a 24 h work shift also confirmed the presence of acute physiological stress symptoms in paramedics. Specifically, stress exposure due to emergency calls was reflected in an increased heart rate, as well as lower RMSSD and higher LF/HF Ratio. This pattern thus clearly indicates heightened sympathetic activation and reduced vagal tone which are characteristic of a stress situation (Karlsson et al., 2011[32]). 

Previous research assessing HRV during work shifts in other professions reported an association of HRV parameters with psychological stress measures indicating that low RMSSD and high LF/HF-Ratio are predictive of higher subjectively experienced work-related stress (Collins and Karasek, 2010[14]; Clays et al., 2011[12]; Karlsson et al., 2011[32]; Hernandez-Gaytan et al., 2013[30]; Borchini et al., 2015[4]). In line with that, we also found that higher heart rate reactions during emergency are related to reduced detachment from work and with higher scores on the PSQ subscale “demands”. Furthermore, work related flow was higher in participants exhibiting less stress during the shift as indicated by lower LF/HF-Ratio. However, associations with other more obvious psychological stress measures failed to reach significance. Although a lack of power due to the small sample size needs to be acknowledged, these findings may indicate that less explicit psychological measures such as work demands or detachment are more likely to reflect paramedics' stress burden.

Finally, we compared paramedics with white-collar workers regarding their coping strategies. Our findings here are also in line with the observed discrepancy between the psychological and the physiological stress parameters in paramedics compared to controls: more often than white-collar workers they used the strategies of denial and positive reframing. Also, paramedics showed higher values of depersonalization - a facet of burnout, which could also be understood as a coping strategy to deal with traumatic events. However, not being aware of one's own stress reaction might lead to insufficient stress-management and self-care (Brosschot, 2010[5]). This could be one reason for the fact that paramedics have an increased risk for burnout (Reardon et al., 2020[42]) and are more likely to quit their job (Chapman et al., 2009[10]). Accordingly, effective interventions should be developed that address the awareness of one's own stress reaction as well as strategies that tackle rescue service specific stressors and physiological stress reactions.

As a limitation of our study we need to acknowledge that due to the sample size we lacked the power to detect small to medium-sized effects. Further studies with larger samples need to be conducted in the future for deeper insights into this important topic. 

In conclusion, this study analyzed and compared many different psychological and physiological stress parameters in paramedics. Despite the high-perceived stress during rescue operations, alteration of heart rate, higher cortisol levels and higher levels of proinflammatory cytokines, paramedics tend to deny their psychological strain. Therefore, questionnaires alone are not sufficient to measure physical stress perception. Moreover, stress denial can lead to an increase in stress-related disease and burnout. Therefore, the paramedic-specific stressors should be addressed in target group specific trainings and coaching. Aim of those trainings is to learn to recognize and to handle these stressors in order to assure employability and psychological health. Accordingly, future research should also address paramedic-specific resources that can be implemented in order to counter work-related adversities. 

## Notes

Corinna Peifer, Vera Hagemann and Maren Claus contributed equally as first authors. 

## Acknowledgements

We thank the ASB group and all the participants of the study for investing their time and make this study possible at all.

We would also like to thank Mrs. Gabi Baumhoer and Dr. Meinolf Blaszkewicz for the analysis of the saliva samples.

## Funding

This research did not receive any specific grant from funding agencies in the public, commercial, or not-for-profit sectors.

## Competing interests

The authors declare that they have no conflict of interest.

## Supplementary Material

Supplementary information

## Figures and Tables

**Table 1:**
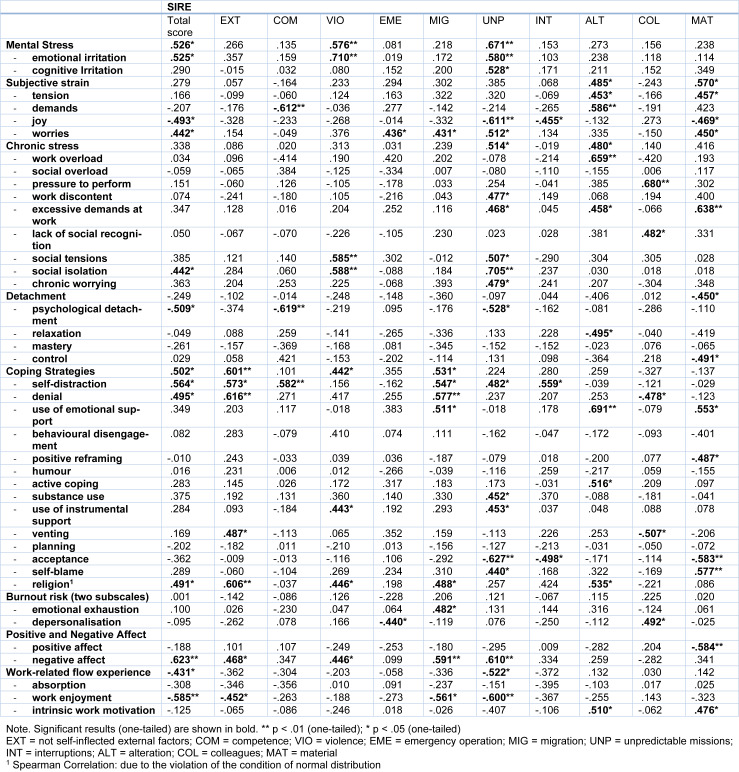
Pearson correlation of the rescue service specific stressors (SIRE) and its subscales with other psychological variables. Statistical tests used are indicated in the table.

**Table 2:**
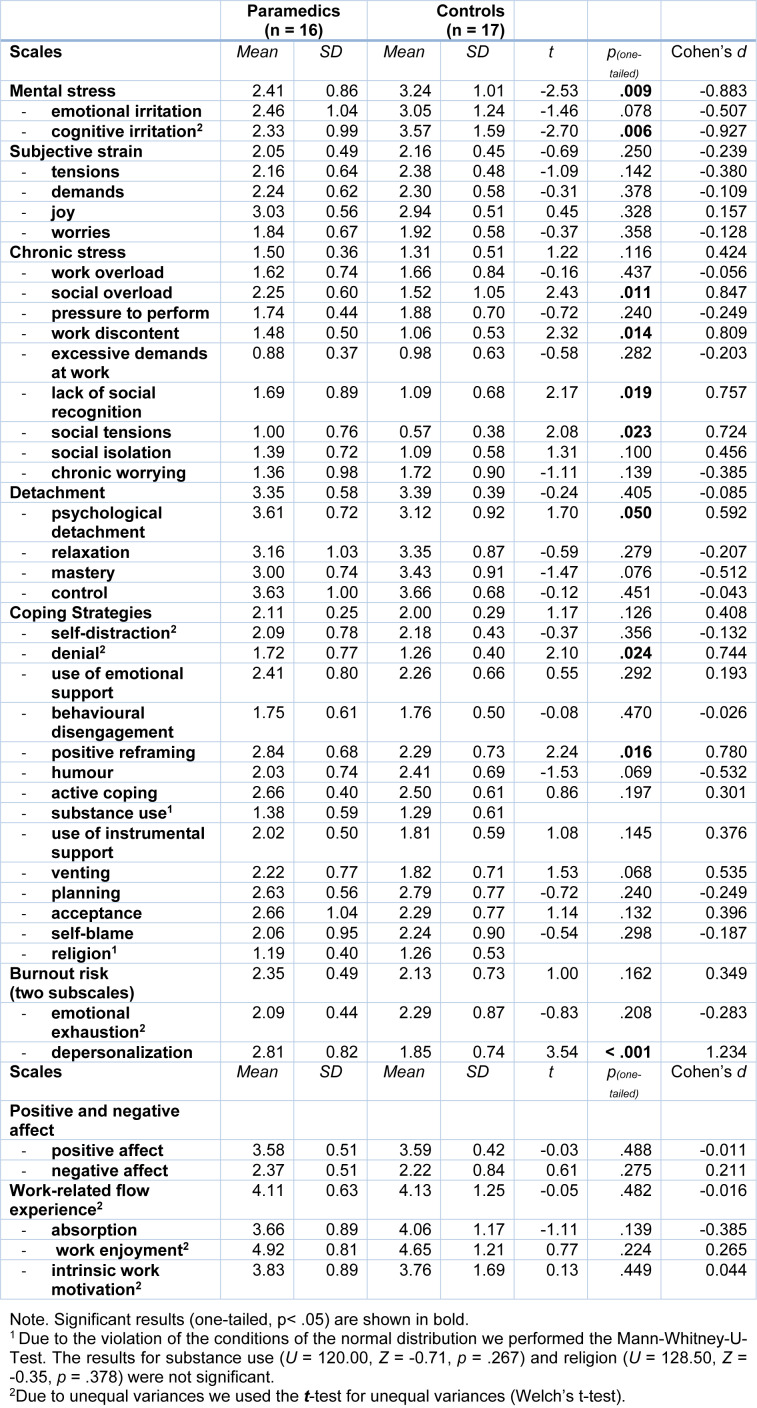
Psychological stress parameters in paramedics and control. Means, standard deviations, significance test and effect sizes of paramedics and white-collar controls are shown.
