# Supplementary material for: Low self-reported stress despite immune-physiological changes in paramedics during rescue operations
Source: EXCLI J. 2021 Apr 16;20:792–811. doi: 10.17179/excli2021-3617 (PMC8073856; doi:10.17179/excli2021-3617)
Supplement: Supplementary information [file EXCLI-20-792-s-001.pdf]

*EXCLI Journal 2021;20:792-811 – ISSN 1611-2156*
*Received: March 11, 2021, accepted: April 07, 2021, published: April 16, 2021*

## Supplementary information to:

## Original article:

# LOW SELF-REPORTED STRESS DESPITE IMMUNE-PHYSIOLOGICAL CHANGES IN PARAMEDICS DURING RESCUE OPERATIONS

Corinna Peifer[1#], Vera Hagemann[2#], Maren Claus[3#], Mauro F. Larra[4], Fabienne Aust[1,5], Marvin Kühn[5], Monika Owczarek[5], Peter Bröde[3], Marlene Pacharra[4,6], Holger Steffens[7], Carsten Watzl[3], Edmund Wascher[4], Silvia Capellino[3,*]

[1] University of Lübeck, Department of Psychology, Lübeck, Germany
[2] University of Bremen, Faculty of Business Studies and Economics, Bremen, Germany
[3] Leibniz Research Centre for Working Environment and Human Factors (IfADo), Department of Immunology, Dortmund, Germany
[4] IfADo- Leibniz Research Centre for Working Environment and Human Factors (IfADo), Department of Ergonomics, Dortmund, Germany
[5] Ruhr University Bochum, Faculty of Psychology, Bochum, Germany
[6] MSH Medical School Hamburg, University of Applied Sciences and Medical University, Hamburg, Germany
[7] Arbeiter-Samariter-Bund, Dortmund, Germany

[#] These authors contributed equally to this work.

* **Corresponding author:** Silvia Capellino, PhD, Neuroimmunology Group, Department of Immunology, Leibniz Research Centre for Working Environment and Human Factors (IfADo), Ardeystrasse 67, 44139 Dortmund, Germany. Tel +49 231 1084 420, E-mail: capellino@ifado.de

http://dx.doi.org/10.17179/excli2021-3617

This is an Open Access article distributed under the terms of the Creative Commons Attribution License (http://creativecommons.org/licenses/by/4.0/).

## SUPPLEMENTARY INFORMATION

Detailed description of the self-report questionnaires:

*1. Stress from rescue service specific stressors* was measured with the 19-item "Stress during rescue service mission Scale" (SIRE)[1] on a 5-point rating scale (1 = *very little* to 5 = *very high*). The scale consists of six subscales named *competence* (4 items), *violence* (3 items), *not self-inflicted external factors* (4 items), *emergency operation* (3 items), *migration* (2 items), and *unpredictable missions* (3 items). Seven items were added to the scale belonging to the subscales *interruptions* (3 items), *colleagues* (2 items), *alteration* (1 item) and *material* (1 item). The whole scale consists of 26 items. An example item is "My perceived stress during rescue service missions with patients, who penetrate my personal safe area, is … ". Coefficient alpha for the whole scale was .82 and it ranged between .41 and .81 for the subscales. The SIRE was only filled in by the paramedics.

*2. Mental Stress* was assessed with the 8-item Irritation Scale[2] on a 7-point rating scale (1 = *not at all* to 7 = *almost totally true*). The scale consists of the two dimensions *emotional irritation* (5 items, e.g., "I get grumpy when others approach me.") and *cognitive irritation* (3 items, e.g., "Even at home I often think of my problems at work."). Coefficient alpha for the whole scale was .80 and the subscales were .83 (*emotional irritation*) and .86 (*cognitive irritation*).

*3. Subjective strain* was assessed with the German version[3] of the Perceived Stress Questionnaire (PSQ)[4] on a 4-point rating scale (1= *hardly never* to 4 = *mostly*). The questionnaire consists of 20 items – four subscales with 5 items each. The subscales are named *tension* (e.g., "You feel tense"), *demands* (e.g., "You have too many things to do"), *joy* (e.g., "You are full of energy"), and *worries* (e.g., "You feel frustrated"). The coefficient alpha were .90 for the whole scale, .81 (*tension*), .76 (*demands*), .80 (*joy*) and .77 (*worries*).

*4. Chronic stress* was assessed with the Trier Inventory of Chronic Stress (TICS)[5]. The inventory consists of 57 items that are grouped into nine subscales named *work overload* (8 items), *social overload* (6 items), *pressure to perform* (9 items), *work discontent* (8 items), *excessive demands at work* (6 items), *lack of social recognition* (4 items), *social tensions* (6 items), *social isolation* (6 items) and *chronic worrying* (4 items). An example item is "I have too many tasks to perform". The chronic stress was measured on a 5-point rating scale (0 = *never* to 4 = *very often*). The coefficient alpha of the whole scale was .93 and it ranged between .77 and .90 for the subscales.

*5. Detachment* was measured with the 16-item Recovery Experience Questionnaire[6] on a 5-point rating scale (1 = *I do not agree at all* to 5 = *I totally agree*). The questionnaire consists of four subscales named *psychological detachment* (e.g., "During time after work I forget about

---

[1] Hagemann V, Holtz M. Entwicklung eines web-basierten Fragebogens zur Messung von "Stress im Rettungsdiensteinsatz - SIRE". Wirtschaftspsychologie. 2016;18(1):78-90.

[2] Mohr G, Rigotti T, Müller A. Instrumente der Arbeits-und Organisationspsychologie. Irritation - ein Instrument zur Erfassung psychischer Beanspruchung im Arbeitskontext. Skalen-und Itemparameter aus 15 Studien. Z Arb & Organisationspsychol A&O. 2005;49:44-8.

[3] Fliege H, Rose M, Arck P, Levenstein S, Klapp B. Validation of the" Perceived Stress Questionnaire"(PSQ) in a German sample. Diagnostica. 2001;47:142-52.

[4] Levenstein S, Prantera C, Varvo V, Scribano ML, Berto E, Luzi C, et al. Development of the Perceived Stress Questionnaire: a new tool for psychosomatic research. J Psychosom Res. 1993;37:19-32.

[5] Schulz P, Schlotz W, Becker P. Trierer Inventar zum chronischen Stress: TICS. Göttingen: Hogrefe, 2004.

[6] Sonnentag S, Fritz C. The Recovery Experience Questionnaire: development and validation of a measure for assessing recuperation and unwinding from work. J Occup Health Psychol. 2007;12:204-21.

EXCLI Journal 2021;20:792-811 – ISSN 1611-2156
Received: March 11, 2021, accepted: April 07, 2021, published: April 16, 2021

work"), *relaxation* (e.g., "During time after work I do relaxing things"), *mastery* (e.g., "During time after work I do things that challenge me") and *control* (e.g., "During time after work I decide my own schedule"). Each subscale includes 4 items. Coefficient alpha for the whole scale was .80. For the subscales coefficient alpha were .91 (*psychological detachment*), .89 (*relaxation*), .93 (*mastery*) and .90 (*control*).

*6. Coping Strategies* were measured with 28 items on a 4-point rating scale (1 = *not at all* to 4 = *to a great extend*) based on the German Brief [7] COPE (Coping Orientation to Problems Experienced)[8]. There are 13 subscales with 2 items (*acceptance, denial, positive reframing, behavioural disengagement, substance use, self-distraction, use of emotional support, humour, active coping, venting, planning, self-blame, religion*) and the subscale *use of instrumental support* that includes 4 items. To specify the subscale *use of instrumental support* we divided between professional advice, private advice and advices from colleagues, so that we had in total 30 items. An example item is "I've been making jokes about it.". The coefficient alpha for the whole scale was .73 and it ranged between .31 and .92.

*7. Burnout risk* was measured with the German version[9] of the Maslach Burnout Inventory (MBI)[10]. The two subscales *emotional exhaustion* (EE, e.g., "Working with people all day is really a strain for me") and *depersonalisation* (DE, e.g., "I don't really care much about what happens to some patients") were used. These subscales consist of 14 items (9 for EE and 5 for DE) and were measured on a 6-point rating scale (1 = *not at all* to 6 = *very often*). The coefficient alpha was .81. For the subscale EE the coefficient alpha was .82 and for the subscale DE it was .74. An example item is "I feel used up at the end of the workday".

*8. Positive and Negative Affect* were measured with a German version[11] of the Positive and Negative Affect Schedule (PANAS)[12] with 9 items for *positive affect* and 7 items for *negative affect*. The 5-point rating scale ranged from 1 (*not at all*) to 5 (*totally true*) and examples of the adjectives, which had to be rated, are interested or upset. The coefficient alpha were .76 for *positive affect* and .79 for *negative affect.*

*9. Work-related flow experience* was assessed with the 13-item Work-Related Flow Inventory (WOLF)[13] on a 7-point rating scale (1 = *never* to 7 = *always).* The inventory consists of three subscales named *absorption* (e.g., "I get carried away by my work"), *work enjoyment* (e.g., "My work gives me a good feeling") and *intrinsic work motivation* (e.g. "I work because I enjoy it"). The coefficient alpha for the whole scale was .91. For the subscale *absorption* (4 items) the coefficient alpha was .81, for *work enjoyment* (4 items) the coefficient alpha was .92 and for *intrinsic work motivation* (5 items) the coefficient alpha was .85.

[7] Knoll N, Rieckmann N, Schwarzer R. Coping as a mediator between personality and stress outcomes: A longitudinal study with cataract surgery patients. Eur J Pers. 2005;19:229-47.

[8] Carver CS, Scheier MF, Weintraub JK. Assessing coping strategies: A theoretically based approach. J Pers Soc Psychol. 1989;56:267-83.

[9] Büssing A, Perrar K-M. Die Messung von Burnout. Untersuchung einer deutschen Fassung des Maslach Burnout Inventory (MBI-D). Diagnostica. 1992;38: 328-53.

[10] Maslach C, Jackson SE. The measurement of experienced burnout. J Organiz Behav. 1981;2:99-113.

[11] Krohne HW, Egloff B, Kohlmann C-W, Tausch A. Untersuchungen mit einer deutschen Version der "Positive and negative Affect Schedule" (PANAS). Diagnostica. 1996;42:139-56.

[12] Watson D, Clark LA, Tellegen A. Development and validation of brief measures of positive and negative affect: the PANAS scales. J Pers Soc Psychol. 1988;54: 1063-70.

[13] Bakker AB. The work-related flow inventory: Construction and initial validation of the WOLF. J Vocat Behav. 2008;72:400-14.

*EXCLI Journal 2021;20:792-811 – ISSN 1611-2156*
*Received: March 11, 2021, accepted: April 07, 2021, published: April 16, 2021*

**Supplementary Table 1: Scales, number of items and coefficient alpha**

| Scale | | Items | Coefficient Alpha |
|---|---|---|---|
| SIRE | | 26 | .82 |
| | competence | 4 | .58 |
| | violence | 3 | .81 |
| | not self-inflicted external factors | 4 | .81 |
| | emergency operation | 3 | .47 |
| | migration | 2 | .41 |
| | unpredictable missions | 3 | .68 |
| | interruptions | 3 | .54 |
| | colleagues | 2 | .46 |
| | alteration | 1 | |
| | material | 1 | |
| Irritation Scale | | 8 | .80 |
| | emotional irritation | 5 | .83 |
| | cognitive irritation | 3 | .86 |
| PSQ | | 20 | .90 |
| | tension | 5 | .81 |
| | demands | 5 | .76 |
| | joy | 5 | .80 |
| | worries | 5 | .77 |
| TICS | | 57 | .93 |
| | work overload | 8 | .90 |
| | social overload | 6 | .87 |
| | pressure to perform | 9 | .78 |
| | work discontent | 8 | .77 |
| | excessive demands at work | 6 | .79 |
| | lack of social recognition | 4 | .84 |
| | social tensions | 6 | .85 |
| | social isolation | 6 | .77 |
| | chronic worrying | 4 | .87 |
| Recovery Experience Questionnaire | | 16 | .80 |
| | psychological detachment | 4 | .91 |
| | relaxation | 4 | .89 |
| | mastery | 4 | .93 |
| | control | 4 | .90 |
| COPE | | 30 | .73 |
| | acceptance | 2 | .88 |
| | use of instrumental support | 4 | .61 |
| | denial | 2 | .63 |
| | positive reframing | 2 | .77 |
| | behavioural disengagement | 2 | .31 |
| | substance use | 2 | .92 |
| | self-distraction | 2 | .38 |
| | use of emotional support | 2 | .62 |
| | humour | 2 | .60 |
| | active coping | 2 | .37 |
| | venting | 2 | .55 |
| | planning | 2 | .51 |
| | self-blame | 2 | .83 |
| | religion | 2 | .33 |
| MBI (two subscales) | | 14 | .81 |
| | emotional exhaustion | 9 | .82 |
| | depersonalisation | 5 | .74 |
| PANAS | | | |
| | positive affect | 9 | .76 |
| | negative affect | 7 | .79 |
| WOLF | | 13 | .91 |
| | absorption | 4 | .81 |
| | work enjoyment | 4 | .92 |
| | intrinsic work motivation | 5 | .85 |

EXCLI Journal 2021;20:792-811 – ISSN 1611-2156
Received: March 11, 2021, accepted: April 07, 2021, published: April 16, 2021

**Supplementary Table 2: Tables of antibodies used for flow cytometry**

| panel | antigen | clone | fluorochrome | company | dilution 1/x |
|---|---|---|---|---|---|
| absolute cell count | CD19 | HIB19 | BV421 | BD Horizon™ | 100 |
| | CD3 | UCHT1 | BV510 | BD Horizon™ | 100 |
| | CD66b | G10F5 | BB515 | BD Horizon™ | 100 |
| | CD56 | B159 | PerCP-Cy5.5 | BD Pharmingen™ | 100 |
| | CD14 | MφP9 | PE | BD Pharmingen™ | 100 |
| | CD45 | HI30 | AF700 | BD Pharmingen™ | 100 |
| (1) general overview of lymphocytes / monocytes | CD19 | HIB19 | BV421 | BD Horizon™ | 200 |
| | CD3 | UCHT1 | BV510 | BD Horizon™ | 400 |
| | live / dead | | zombie Yellow | Biolegend | 1000 |
| | CD16 | 3G8 | FITC | BD Pharmingen™ | 200 |
| | HLA-DR | G46-6 | PerCP-Cy5.5 | BD Horizon™ | 100 |
| | CD14 | MφP9 | PE | BD Pharmingen™ | 500 |
| | CD64 | 10.1 | PE-Cy7 | BD Pharmingen™ | 200 |
| | CD56 | B159 | APC | BD Pharmingen™ | 50 |
| | CD45 | HI30 | AF700 | BD Pharmingen™ | 500 |
| (2) memory and homing markers of NK cells and T cells | KLRG1 | 2F1 | BV421 | BD Horizon™ | 400 |
| | CD3 | UCHT1 | BV510 | BD Horizon™ | 400 |
| | live / dead | | zombie Yellow | Biolegend | 1000 |
| | CD8 | RPA-T8 | FITC | BD Pharmingen™ | 200 |
| | CD28 | CD28.2 | PerCP-Cy5.5 | BD Pharmingen™ | 100 |
| | CD57 | NK-1 | PE | BD Pharmingen™ | 800 |
| | CD56 | B159 | PE-CF594 | BD Pharmingen™ | 100 |
| | CD62L | DREG-56 | PE-Cy7 | BD Horizon™ | 200 |
| | CD197 | 150503 | AF647 | BD Pharmingen™ | 50 |
| | CD4 | RPA-T4 | APC-H7 | BD Pharmingen™ | 100 |
| | CD45RA | HI100 | AF700 | BD Pharmingen™ | 400 |
| (3) activation and memory markers of NK cells and T cells | KLRG1 | 2F1 | BV421 | BD Horizon™ | 400 |
| | CD3 | UCHT1 | BV510 | BD Horizon™ | 400 |
| | live / dead | | zombie Yellow | Biolegend | 1000 |
| | NKG2C | 134591 | AF488 | R&D Systems | 100 |
| | CD56 | B159 | PerCP-Cy5.5 | BD Pharmingen™ | 50 |
| | CD57 | NK-1 | PE | BD Pharmingen™ | 800 |
| | CD25 | M-A251 | PE-Cy7 | BD Pharmingen™ | 50 |
| | DNAM-1 | DX11 | AF647 | BD Pharmingen™ | 100 |
| | CD69 | FN50 | APC-H7 | BD Pharmingen™ | 100 |

*Received: March 11, 2021, accepted: April 07, 2021, published: April 16, 2021*

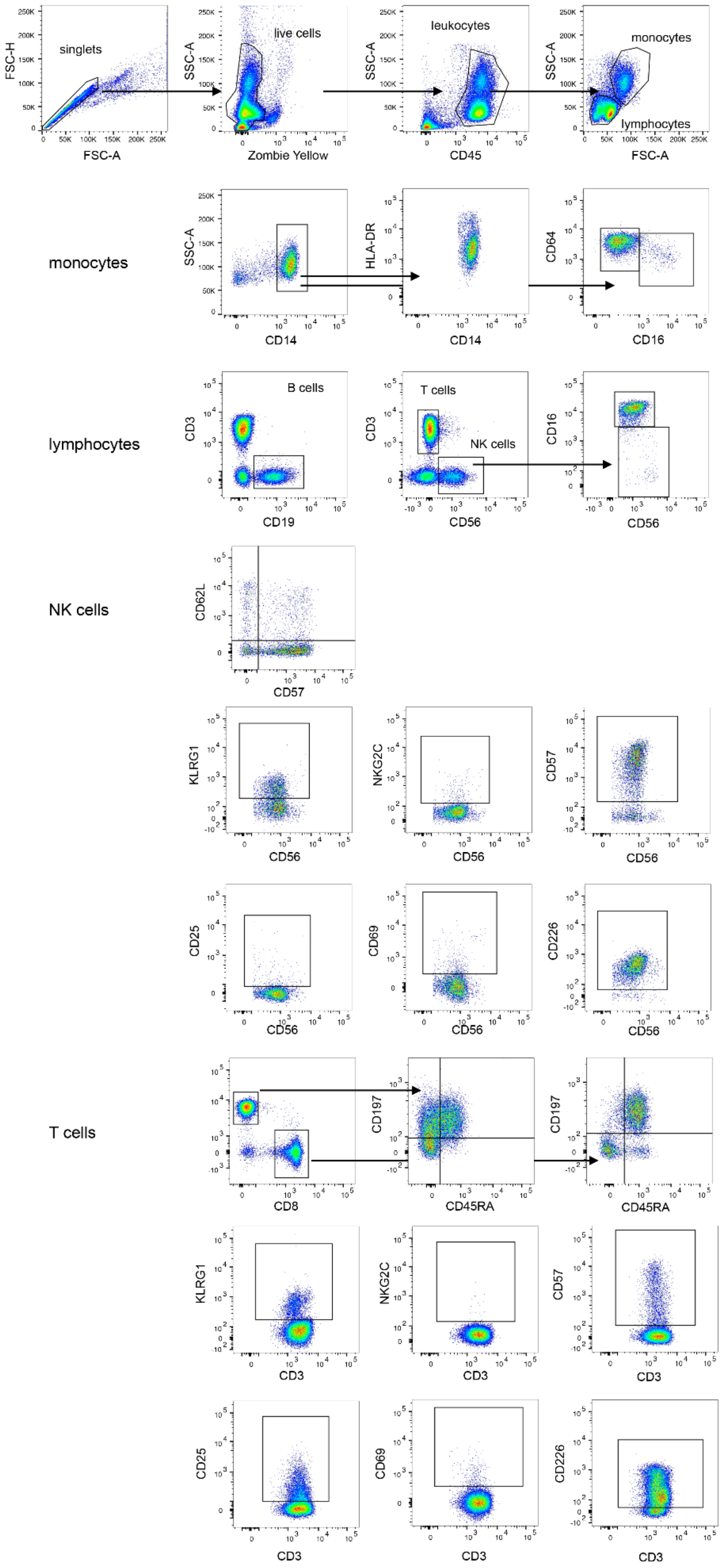


**Supplementary Figure 1:** Gating strategy used for FACS analysis

EXCLI Journal 2021;20:792-811 – ISSN 1611-2156
Received: March 11, 2021, accepted: April 07, 2021, published: April 16, 2021

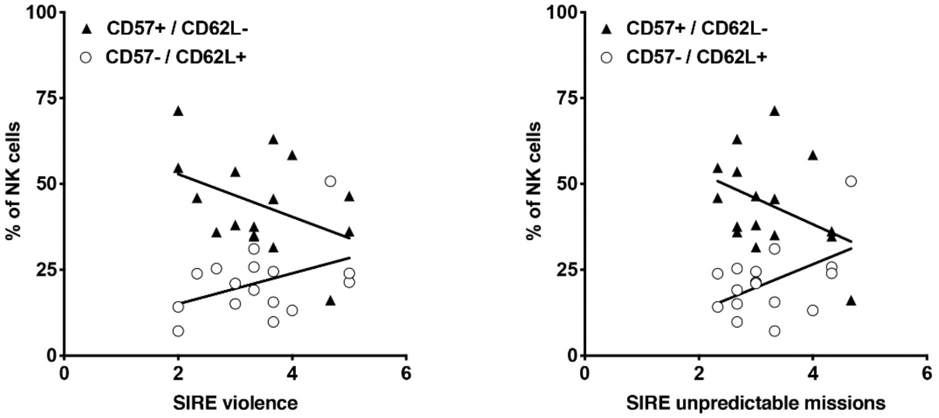


**Supplementary Figure 2:** Correlation of paramedics NK cell subsets with the SIRE subscales "violence" and "unpredictable missions"
